# Utilization of Spent Coffee Grounds as an Antioxidant Dietary Fiber in Beef Patties: Oxidative Stability, Texture Properties, and Molecular Docking

**DOI:** 10.1002/fsn3.70919

**Published:** 2025-10-03

**Authors:** Nazik Meziyet Dilek

**Affiliations:** ^1^ Department of Nutrition and Dietetics, Akşehir Kadir Yallagöz School of Health Selçuk University Konya Turkey

**Keywords:** antioxidant dietary fiber, beef, human nutrition, molecular docking, spent coffee grounds

## Abstract

Spent coffee grounds (SCG) are rich in valuable nutrients, including dietary fiber, proteins, lipids, vitamins, minerals, and various bioactive compounds. In the context of food science, it is essential to reconsider SCG not as waste, but as a potential functional ingredient that can be valorized to reduce environmental impact and enhance nutritional and economic value in food systems. In this study, SCG of Turkish, espresso, or filter coffee was evaluated as a resource of dietary fiber and antioxidants in beef patties during refrigerated storage over 7 days. Physicochemical (pH, water activity, color, cooking loss, diameter reduction, dietary fiber content, and oxidative stability), textural, and sensory traits of beef patties were determined. The spent coffee ground treatment significantly increased the dietary fiber content of the samples (*p* < 0.05). The lowest TBARS number was found in the groups of treatment (T1, T2, and T3) on the 7th day. Additionally, the pharmacokinetic properties and safety profiles of selected SCG‐derived phytochemicals were assessed using ADMET analysis, revealing favorable bioavailability and non‐inhibitory effects on cytochrome P450 enzymes. Molecular docking studies against the 2FLU (Kelch‐Neh2 Complex) target protein demonstrated strong binding interactions, with chlorogenic acid exhibiting the highest affinity (−9.3 kcal/mol, Ki: 0.152 μM). The sensory scores showed that incorporating SCG, especially Turkish and filter coffee grounds, improved the odor properties of beef patties but adversely affected texture due to their fibrous and porous nature. Yet, general consumer acceptability was higher for these samples compared to the control group. As a result, utilizing SCG as a resource of antioxidants and dietary fiber in meat products represents a viable, natural, and cost‐effective solution for the meat industry.

## Graphical abstract

SCG enhanced the dietary fiber content and provided antioxidant protection against lipid oxidation in beef patties during storage. Molecular docking results also confirmed strong interactions, especially for chlorogenic acid, supporting the potential of SCG as a functional and sustainable ingredient in meat products.

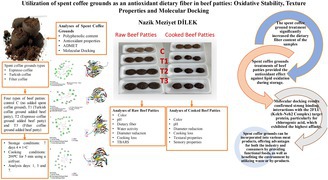

## 1 Introduction

For centuries, protein‐rich foods have been fundamental in human diets across civilizations and remain so in many societies today. Meat products are a nutritious source containing high‐quality protein, fat, vitamins (especially B group vitamins), and minerals (such as iron, zinc, selenium, and phosphorus) (Libera et al. 2021). Recent studies indicate that meat products, which are increasingly recognized for their significance in nutrition, health, economy, and social structure amid global population growth, have been found to exert both positive and negative effects on health (Geiker et al. 2021). Several extensive observational researches have identified significant links between increased consumption of red and processed meats and elevated risks of cardiovascular diseases, cancer, all‐cause mortality, and type 2 diabetes (Pan et al. 2012; Wang et al. 2016). The adverse effects of these products on health are associated with factors such as high saturated fat and cholesterol content, preservatives like nitrites and nitrates, carcinogens such as heterocyclic amines and polycyclic aromatic hydrocarbons formed during improper cooking processes, high salt, and low fiber content (Adeyeye 2018; Bernardo et al. 2021; Das et al. 2020; Delgado et al. 2021). In recent years, research has increasingly focused on the health effects of meat products, particularly their association with long‐term health issues such as obesity, cardiovascular diseases, and diabetes. At the same time, innovative approaches are being developed to improve the technologies used in the production processes of meat products and to increase product quality, safety, and storage stability. It offers solutions on sustainability‐oriented research, effective use of resources, and waste management. In order to meet the demands of consumers, it is important to develop processed meat products enriched with new and functional ingredients and to examine the potential impacts of these components on health. Over the past years, significant studies aiming at improving the healthiness of meat products have been conducted. Within this scope, efforts have been made to reduce fat (Kumar 2021), salt (Gómez‐Salazar et al. 2021), and preservative/additive usage, while enriching products with functional components such as dietary fiber (Unal et al. 2022) and antioxidants (Babaoğlu et al. 2022; Nitin Mehta et al. 2013).

Coffee is a very old beverage dating back to the 10th century and its consumption has increased significantly all over the world. One of the most important reasons for this is its unique sensory and pleasant flavor as well as that coffee is considered a functional food thanks to the bioactive components such as phenolic components, diterpenes, methylxanthines, nicotinic acid, and trigonelline, magnesium, and potassium it contains (de Melo Pereira et al. 2019).

Coffee's bioactive components help to lower the risk of developing a number of diseases, including type 2 diabetes, depression, suicidal thoughts and behaviors, cancer, liver damage, cirrhosis, and neurological and cardiovascular disorders. Regular coffee consumption also improves mental alertness, has positive effects on the gut microbiota, and helps to eliminate various gastrointestinal system disorders. (de Melo Pereira et al. 2020; Jeszka‐Skowron et al. 2015).

Melanoidins are compounds formed at the final stage of the Maillard reaction, which occurs during the heating of foods containing reducing sugars and amino acids. They area primarily responsible for the characteristic brown color observed in foods such as coffee, bread, and malt (Mesías and Delgado‐Andrade 2017). Various agricultural by‐products, including spent coffee grounds (Arya et al. 2022; Jiménez‐Zamora et al. 2015), distilled spent grain (Yang, Fan, and Xu 2019; Yang, Lou, et al. 2019), and sugarcane molasses (Mikami and Murata 2015), contain varying amounts of underutilized melanoidins. Melanoidins not only contribute to the brown coloration of foods, but also enhance their desirable texture and aroma, thereby positively influencing consumer perception of product quality. For this reason, interest in melanoidins has been steadily increasing across different sectors. They have been reported as promising ingredients for functional beverages (Richelle et al. 2001), bakery products (Martinez‐Saez et al. 2017), dietary supplements, and processed meat products (Lin et al. 2015). In addition to their sensory contributions, melanoidins have been shown to exhibit various biological activities similar to other natural pigments (Langner and Rzeski 2014). They possess antioxidant and antimicrobial properties, support gut health by promoting the growth of beneficial bacteria in the colon, and exhibit anti‐inflammatory effects (Argirova et al. 2013; Mesías and Delgado‐Andrade 2017). Studies have shown that coffee melanoidins offer exciting opportunities for incorporation into functional foods and beverages targeting chronic inflammation management, cancer prevention, and overall digestive health (Argirova et al. 2013). However, to fully understand these newly discovered benefits and to maximize their effects without compromising sensory properties in various food applications, further research is needed. This includes optimizing the extraction methods, determining appropriate usage levels, and evaluating the safety and bioavailability of these pigments for human consumption.

The most recent preliminary forecast for total consumption in the coffee year of 2023/24 is 177.0 in thousand 60‐kg bags, a 2.2% increase compared to 173.1 in thousand 60‐kg bags in the prior year (International Coffee Organization 2020). The world population is forecasted to attain 8.6 billion by 2030 and 9.8 billion by 2050, with one third of the total populace concentrated in city environments. This is expected to have an impact on coffee production and consumption growth (Hoseini et al. 2021). As a natural consequence of the increase in coffee consumption, it is inevitable that the by‐products of coffee such as spent coffee grounds, defective and premature coffee beans, silverskin, coffee pulp, and coffee husk production and preparation will also increase. Unless these by‐products are reproduced in food and other industries, they pose a major environmental problem for producing countries (Iriondo‐DeHond et al. 2019). On the other hand, utilizing the coffee by‐products as components of natural, healthful foods or as raw materials can contribute to the economy, enrich food products, meet the increasing consumer demand for natural and healthy products, and mitigate their negative environmental impacts (Laufenberg et al. 2003). By‐products of coffee processing are abundant in nutrients including carbohydrates, vitamins, proteins, minerals, dietary fibers, lipids, and bioactive elements including polyphenols and other functional substances (Janissen and Huynh 2018).

The SCG used in this study is a by‐product of coffee preparation methods, including both homemade brewing and coffee machines, as well as indirect methods such as instant coffee production and beverage manufacturing. These grounds have a dark brown color, a coarse texture, and high moisture content (Schwan and Fleet 2014). The primary components of SCG include polysaccharides, which through thermal hydrolysis can yield mannooligosaccharides (MOS). MOS derived from spent coffee exhibit a prebiotic effect by resisting digestion and promoting the growth of bifidobacteria, known to support intestinal health. Moreover, MOS have shown other health advantages like lowered blood pressure and decreased body fat and aiding in protection against oxidative stress‐related diseases like cancer. Spent coffee is increasingly recognized as a natural source of various beneficial compounds including prebiotics, carbohydrates, dietary fiber, and antioxidants (Bravo et al. 2013). It has 2% lipid content, of which 35% is extractable fat and is made up of palmitic and linoleic acids. This by‐product is a rich source of vitamin E with antioxidant qualities because regular espresso coffee and other coffee makers can only extract 1% and 5% of the vitamin E in coffee, respectively (Alves et al. 2017). In recent years, there has been a growing interest in incorporating spent coffee grounds (SCG) directly into food formulations due to their high content of dietary fiber, phenolic compounds, and antioxidant potential. Various studies have demonstrated the feasibility of using raw or minimally processed SCG as an ingredient in bakery products such as cookies, muffins, and biscuits, with promising nutritional and functional outcomes (Martinez‐Saez et al. 2014, 2017). For instance, SCG‐enriched muffins and shortbread biscuits were found to have improved antioxidant activity, fiber content, and acceptable sensory profiles, even when used at relatively high concentrations. Furthermore, the valorization of SCG in food systems supports sustainability goals by reducing food waste and promoting circular economy practices in the food industry (Mussatto et al. 2011). Despite some challenges regarding bitterness or color, recent work continues to refine formulations to enhance consumer acceptance and functionality.

Molecular docking has been recognized as an effective method in clinical research, sequence analysis platforms, and molecular modeling. To streamline the complex and multi‐step process of novel drug discovery, in silico techniques such as molecular docking are commonly employed (Hasan et al. 2022). Docking is a computational approach that evaluates various conformations of small molecules within the binding sites of target proteins, using scoring functions to determine the best‐fitting conformation. Molecular docking is widely used for identifying lead compounds and plays a critical role in computer‐aided drug design and structural molecular biology. The primary objective of ligand–protein docking is to predict the most favorable binding modes between ligands and their target proteins (Singh et al. 2023). Molecular docking can be utilized to assess the therapeutic potential of a compound at an early stage, thereby saving time and reducing costs in the drug development process. Incorporating ADMET (absorption, distribution, metabolism, excretion and toxicity) parameters into this evaluation allows for a more comprehensive assessment of the compound's safety profile and efficacy (Cho et al. 2024).

In recent years, molecular docking has been increasingly applied not only in drug discovery but also in the field of food science to investigate the bioactivity of food‐derived compounds. This in silico technique enables the prediction of interactions between bioactive food molecules—such as polyphenols, peptides, and alkaloids—and biological targets like enzymes and receptors. Such predictions help assess potential health benefits, including antioxidant, anti‐inflammatory, and anticancer properties (Tao et al. 2020; Vidal‐Limon et al. 2022; Aarón et al. 2025). Molecular docking also contributes to the design of functional foods and nutraceuticals by identifying promising compounds with strong binding affinities to health‐related targets (Carpio et al. 2021). Additionally, it aids in evaluating food‐drug interactions and the allergenic potential of food proteins (Nevado‐Bulnes et al. 2024). Coffee‐derived melanoidins and phenolics have also been studied using molecular docking to understand their antioxidant mechanisms (Aytar and Aydın 2025). Despite its advantages, experimental validation remains essential to confirm docking results. Nevertheless, molecular docking offers a valuable, cost‐effective screening tool for guiding food bioactivity research and innovation.

In a study, the effects of *Epilobium angustifolium* (willow herb) extract on the quality of beef burgers during refrigerated storage were investigated. The extract showed notable antioxidant activity, supported by high total phenolic and flavonoid contents. GC–MS and HPLC analyses identified several bioactive compounds, including α‐pinene, β‐thujone, ascorbic acid, and gallic acid. Molecular docking revealed that gallic acid, β‐thujone, and α‐pinene interact with 9R‐lipoxygenase, indicating potential antioxidant mechanisms. These findings highlight the extract's potential as a natural preservative in meat products (Dilek et al. 2025).

In a study examining the chemical composition and antioxidant properties of coffee beans at various roasting stages, molecular docking techniques were employed to evaluate the interactions between coffee‐derived compounds and biological targets associated with antioxidant activity. The results suggest that roasting level has a significant influence on the structure and bioactivity of coffee's health‐promoting constituents (Aytar and Aydın 2025).

The potential of freeze‐dried black currant leaf extract as a natural antioxidant and bioactive additive in canned pork meat, particularly in formulations with reduced sodium nitrite content, was investigated. The researchers assessed both the antioxidant activity and the enzyme‐inhibitory properties of the extract‐fortified meat products. To better understand the mechanism of action of the peptides generated during meat processing, molecular docking analyses were performed. The molecular docking results supported the hypothesis that the extract contributes not only to oxidative stability but also to potential health benefits, confirming its value as a functional ingredient in meat preservation and enhancement (Wójciak and Kęska 2023).

Wang et al. (2023) investigated the preservative efficacy of a composite biopreservative comprising chitosan, tea polyphenols, and additional natural agents on goat meat during chilled storage. Molecular docking analyses were employed to explore the binding affinities of key bioactive compounds to target proteins related to oxidative pathways, offering mechanistic insights into the observed antioxidant effects. Consequently, the researchers suggest that this natural, composite preservative formulation could be a viable alternative to synthetic additives for extending the shelf life and improving the oxidative stability of refrigerated goat meat.

Using Turkish, espresso, or filter coffee grounds, which are by‐products of coffee processing, to reformulate meat products would provide bioactive elements like dietary fiber and antioxidants. This approach could improve their physiological and functional properties, as well as their oxidative stability. Reusing waste products saves resources by contributing to the protection of natural resources and reducing consumption. It also enables waste to contribute to the economy by making it possible to recover economically valuable materials. In addition, it reduces negative impacts on the environment. It helps reduce landfills and environmental pollution. It encourages the transition to sustainable production and consumption models, thus contributing to leaving a healthier environment for future generations. In addition, it is an important step to comply with legal regulations and social responsibility principles on waste management. For these reasons, the reuse of waste products provides significant benefits both environmentally and economically. However, there are no reports on the use of these by‐products as antioxidant dietary fiber in meat products. Thus, the purpose of this study was to evaluate these materials' antioxidant capacity, investigate their effects on the physicochemical, textural, and sensory characteristics of beef patties, and assess the bioactive potential of SCG‐derived compounds through molecular docking. The binding interactions of key phytochemicals with the 2FLU (Kelch‐Neh2 Complex) target protein were analyzed, highlighting their potential functional and health‐related applications.

## 2 Material and Methods

### 2.1 Proximate Content

The moisture, total protein, total fat, total ash, and pH values of the minced beef meat were determined using AOAC (2000) standard methods.

### 2.2 Polyphenolic Content of SCG

The caffeine content in SCG was assessed using the methodology outlined by Franeta et al. (2002) via an HPLC method.

HPLC was utilized to ascertain the Turkish, espresso, and filter SCG extracts' phenolic content. The extracts were put into the brown vial after being run through a 0.45 μm Teflon membrane filter. Then, using an autosampler system of SIL‐10 ce vp, 20 μL of the extracts in the brown vials were injected into the HPLC (Shimadzu Corporation, Kyoto, Japan). Chromatographic separations were carried out using an Eclipse XDB‐C18 column (Agilent USA) (250 9 4.60 mm) with a particle size of 5 μm. Methanol (B) and 3% acetic acid (A) made up the mobile phase. The following binary gradient was used to elute the extracts: 7% was the starting point for the gradient in order to attain 28% B at 20 min, 25% B at 28 min, 30% B at 35 min, 30% B at 50 min, 33% B at 60 min, 42% B at 62 min, 50% B at 70 min, 70% B at 73 min, 80% B at 75 min, 100% B at 80 min, and 7% B at 81 min. At 30, the flow rate was maintained at 0.8 mL/min (Baltacıoğlu et al. 2021).

### 2.3 Antioxidant Properties of SCG

The total phenolic contents of SCG were determined using the method known as Folin–Ciocalteu method as proposed by Yoo et al. (2004). Using a UV–vis spectrophotometer, absorption was measured at 750 nm. The results were represented as milligrams of gallic acid equivalents (GAE) per 100 mL, with gallic acid serving as the benchmark.

In order to determine the total flavonoid content of SCG, measurements were made at 510 nm based on the method reported by Chen and Chen (2011). The results were given as mg catechin equivalent (mg CE/100 mL).

Free radical scavenging activities of Turkish coffee, espresso coffee, and filter SCG were determined by using the DPPH (1,1‐diphenyl‐2‐picrylhydrazyl) method proposed by Lee et al. (1998). For this purpose, the absorbance was recorded at 517 nm using a spectrophotometer (UV‐160 A, UV–Visible Recording Spectrophotometer, Shi‐madzu, Tokyo, Japan). The findings were given as the free radical scavenging activity percentage (%).

### 2.4 Pharmacokinetic and Safety Evaluation of Selected Phytochemicals Through ADMET Analysis

The pharmacokinetic properties and safety profiles of four selected phytochemicals were assessed using ADMET (Absorption, Distribution, Metabolism, Excretion, and Toxicity) analysis. Initially, the chemical structures of the compounds were converted into SMILES format and analyzed using an online ADME prediction tool. The SWISSADME platform was utilized to estimate key pharmacokinetic parameters, including absorption, metabolism, and elimination, along with essential physicochemical properties.

The evaluated ADMET parameters included molar refractivity, topological polar surface area (TPSA), consensus Log Po/w, gastrointestinal (GI) absorption, blood–brain barrier (BBB) permeability, P‐glycoprotein (P‐gp) substrate potential, cytochrome P450 enzyme inhibition profile (CYP1A2, CYP2C19, CYP2C9, CYP2D6, CYP3A4), dermal penetration coefficient (Log Kp), bioavailability score, and synthetic accessibility. These analyses provided a comprehensive pharmacokinetic framework for assessing the feasibility of the selected phytochemicals for pharmaceutical applications (Cheng et al. 2012; Yang, Lou, et al. 2019).

### 2.5 Molecular Docking Analysis

Molecular docking simulations were performed to evaluate the interactions between the selected compounds and the target protein, 2FLU (Crystal Structure of the Kelch‐Neh2 Complex). Prior to docking, all water molecules and cofactors were removed to prevent interference with binding interactions, and polar hydrogen atoms were added using AutoDockTools (ADT) to optimize the protein structures. Ligand structures were obtained from the PubChem database in SDF format and subsequently converted into PDB format using Discovery Studio Visualizer to ensure compatibility with docking procedures.

The active binding site was defined using AutoGrid, with the grid box centered on the target pocket and dimensions set to 40 points in each direction, maintaining a grid spacing of 0.375 Å. Docking simulations were executed with AutoDock Vina, generating ten distinct binding poses per ligand to evaluate their affinity toward the target proteins. The energy range was limited to 9 kcal/mol, and the exhaustiveness parameter was set to 1000 to enhance the precision and reliability of the docking outcomes.

The docking results were analyzed based on key parameters, including binding energy, ligand efficiency (LE), fit quality (FQ), pIC50, and the estimated inhibition constant (Ki). The obtained ligand‐protein conformations and molecular interactions were visualized in both 2D and 3D representations using BIOVIA Discovery Studio Visualizer (BIOVIA, 2019) for further interpretation(Trott and Olson 2010).

### 2.6 Sample Crafting and Cooking Procedure

Beef patty samples were prepared according to the method given by (Babaoğlu et al. 2022). After weighing each item separately, the materials (breadcrumbs, salt, distilled water, and three types of SCG: Turkish, espresso, and filter) were combined and stirred for around 4 min. As demonstrated in Table 1, four types of beef patties were prepared as follows: control C (no added spent coffee ground), T1 (Turkish coffee ground added beef patty), T2 (Espresso coffee ground added beef patty) and T3 (Filter coffee ground added beef patty). Each beef patty dough (for C, T1, T2, and T3) was randomly divided into two groups for raw and cooked analyses, and beef patty samples were obtained from these beef patty doughs. For analyses on cooked samples (cooking loss, diameter reduction, color, pH and texture characteristics), five beef patty samples from each group were cooked before analysis. The patties were cooked at 200°C for 5 min using an air fryer and then cooled to room temperature for analyses.

**TABLE 1:** Formulating beef patties using various spent coffee grounds.

Ingredients (g)	Treatments			
	C	T1	T2	T3
Meat	720.0	720.0	720.0	720.0
Breadcrumbs	86.4	—	—	—
Distilled water	50.4	50.4	50.4	50.4
Salt	7.2	7.2	7.2	7.2
Spent coffee ground	—	86.4	86.4	86.4

Abbreviations: C, Control; T1, Turkish coffee ground added beef patty; T2, Espresso coffee ground added beef patty; T3, Filter coffee ground added beef patty.

For 7 days, the patties were kept at 4°C ± 1°C after being encased in a polyvinyl chloride film. Three patties for each of the four treatments (C, T1, T2, and T3), three storage times (1, 3, and 7), and two independent replications (using comparable production techniques) resulted in a total of 72 beef patties. The days 1, 3, and 7 were used for the analysis of every sample.

### 2.7 Physical Analyses of Samples

Color determination was performed using a chroma meter (CR‐400, Konica Minolta Inc., Osaka, Japan) under illuminant D65, 2° observer, and diffuse/O mode. The samples' lightness (L*), redness (a*), and yellowness (b*) were evaluated.

The pH value of each sample group was measured raw and after cooking on the analysis days (days 1, 3, and 7) before the analyses. A pH meter (Testo 205, Testo, USA) fitted with a pH probe and a thermometer penetration tip was used to measure the pH of each sample. Standard buffer solutions with known pH values (usually pH 4.00, pH 7.00, and pH 10.00) were used to calibrate the pH meter. The electrode was submerged in each buffer solution throughout the calibration process, the readings were allowed to stabilize, and the meter was then adjusted to match the pH of each buffer. During the calibration procedure, temperature adjustment was carried out. Automatic temperature adjustment was used by the pH meter to modify the pH readings according to the temperature of the fluid being tested. Across a range of temperatures, this function guarantees precise pH readings (Lambooij et al. 1999).

Water activity was determined using a water activity system (Testo 650, Germany).

Two spots were marked on each patty to measure the variation in diameter before and after cooking. The patties were cooked at 200°C for 5 min using an air fryer and subsequently cooled to room temperature. A digital caliper was used to measure the diameters of the raw and cooked patties, and the formula was used to determine the % decrease in diameter:

$$\begin{array}{cc} \text{Diameter reduction}\;\left(\%\right)= & \left[\mathrm{Raw}\;\text{patty diameter}-\text{Cooked patty diameter}\right]/\\ & \mathrm{Raw}\;\text{patty diameter}\times 100 \end{array}$$

Likewise, the cooking loss of samples was computed using the following method after the samples were weighed both before and after cooking:

$$\begin{array}{cc} \text{Cooking loss}\;\left(\%\right)= & \left[\text{Weight before cooking}-\text{Weight after cooking}\right]/\\ & \text{Weight before cooking}\times 100 \end{array}$$

### 2.8 TBARS Number of Samples

The TBARS number, which is accepted as an indicator for the determination of lipid oxidation, was measured using a spectrophotometer (UV‐160 A, UV–Visible Recorder Spectrophotometer, Shimadzu, Tokyo, Japan) at 530 nm according to the method given by Tarladgis et al. (1960). The TBARS number was determined as mg malonaldehyde/kg sample by multiplying the absorbance values by the constant 7.03.

### 2.9 Dietary Fiber Composition of Samples

The total dietary fiber composition of beef patty samples was assessed using the dietary fiber assay kit (Megazyme, Wicklow, Ireland) according to the AACC (2000) method. The beef patty samples underwent enzymatic digestion using α‐amylase, protease, and amyloglucosidase sequentially, and the total dietary fiber was quantified as the combined amount of soluble and insoluble dietary fiber.

### 2.10 Textural Traits of Samples

To evaluate the textural characteristics of cooked beef patty samples, a texture analyzer (TA.XT‐Plus, Stable Micro Systems, Godalming, Surrey, United Kingdom) was utilized following the procedure established by Herrero et al. (2007). The patties were cooked at 200°C for 5 min using an air fryer and subsequently cooled to room temperature. Each group included five samples, each of which underwent two compressions.

### 2.11 Sensory Traits of Samples

Sensory evaluations of samples were carried out according to Yıldız‐Turp and Serdaroglu (2010) by a sensory panel consisting of 20 semi‐trained and pre‐informed panelists from Selçuk University, Department of Food Engineering. The samples were served after cooking in the air fryer at 200°C for 5 min. Randomly selected 3‐digit numbers were used to code the samples. Cooked samples were served to the panelists with water and bread to be consumed between samples.

### 2.12 Statistical Analysis

A totally randomized factorial design comprising four treatments (C, T1, T2, and T3) and three storage durations (1, 3, and 7 days) was used in this investigation, which involved two independent replications. The generalized linear mixed model was used to perform analysis of variance (ANOVA) for the statistical study of all parameters. The replication was regarded as a random element, whereas the addition of SCG treatment, storage duration, and their interaction were examined as fixed factors. Using the Tukey Multiple Comparison Test, differences between the means were ascertained at the 5% significance level.

## 3 Results and Discussion

### 3.1 Proximate Content of Minced Beef Meat

The pH, total protein, total fat, moisture, and total ash values of ground meat used in the study were measured as 5.69, 17.90%, 21.49%, 59.76%, and 3.18%, respectively.

The same parameters were reported as 73.41%, 19.17%, 2.35%, 1.09%, and 5.43% for moisture, total protein, total fat, total ash, and pH, respectively, in another study (Erdem et al. 2020).

When certain findings from the literature were compared with the minced beef meat used in the present study, some discrepancies were observed. These differences may be attributed to various factors influencing the chemical composition of meat, including rearing conditions, age, diet, breed, sex, and the specific anatomical location from which the meat was obtained.

### 3.2 Total Phenolic Content and Antioxidant Properties of SCG

Table 2 presents the total phenolic content (TPC), total flavonoid content (TFC), and antioxidant activity (DPPH) of the different wasted coffee ground varieties. Table 3 illustrates the phenolic composition of these varieties. Research has recently looked at the recovery of phenolic compounds and their antioxidant properties from by‐products of the coffee industry. Coffee by‐products such as coffee pulp, husk, silver skin, and spent coffee grounds were used for the extraction of phenolic compounds using a solvent mixture of isopropanol and water. Following viscosim pretreatment, 25% of the total polyphenols were recovered from silver skin, 19% from waste residues, and 17% from cherry husk (Murthy and Naidu 2012).

**TABLE 2:** The antioxidant activity (DPPH), total phenolic (TPC) and flavonoid (TFC) contents of Turkish, Espresso, and Filter coffee grounds.

Spent coffee ground type	DPPH (%)	TPC (mg GAE/100 mL)	TFC (mg CE/100 mL)
Turkish coffee	26.38 ± 3.83^AB^	114.95 ± 6.13^B^	41.16 ± 1.62^A^
Espresso coffee	36.94 ± 2.47^A^	240.96 ± 10.51^A^	40.46 ± 0.93^A^
Filter coffee	13.70 ± 1.03^B^	79.41 ± 1.96^B^	31.67 ± 0.00^B^

*Note:* Mean ± standard error. ^A‐B^Different letters indicate statistically significant differences among means within beef patty samples across storage period (*p* < 0.05).

Abbreviations: C, Control; T1, Turkish coffee ground added beef patty; T2, Espresso coffee ground added beef patty; T3, Filter coffee ground added beef patty.

**TABLE 3:** Polyphenolic compounds of Turkish, Espresso and Filter coffee grounds.

Polyphenolic compound (mg/100 g)	Spent coffee ground type		
	Turkish coffee	Espresso coffee	Filter coffee
Apigenin	< R.L.	< R.L.	< R.L.
Luteolin	< R.L.	< R.L.	< R.L.
Quercetin	< R.L.	< R.L.	< R.L.
Chlorogenic acid	19.4	86.4	< R.L.
p‐coumaric acid	< R.L.	< R.L.	< R.L.
Ferulic‐trans	< R.L.	< R.L.	< R.L.
Rosmarinic acid	< R.L.	< R.L.	< R.L.
Caffeic acid	20.3	48.1	< R.L.
Syringic acid	17.7	71.0	< R.L.
Caffeine	95.0	181.5	6.8

Abbreviation: R.L., Report limit.

Studies have shown that the main phenolic component of coffee is chlorogenic acid. Phenolic compounds, mainly in the form of chlorogenic acid, constitute up to 12% of the solid content of green coffee beans. These water‐soluble esters are formed by the reaction of quinic acid with one or two molecules of caffeic acid, a type of trans‐cinnamic acid. However, it is reported that the amounts of these phenolic compounds will vary depending on the coffee preparation technique (Esquivel and Jimenez 2012).

Phenolic compounds such as apigenin, luteolin, quercetin, p‐coumaric acid, ferulic‐trans, and rosmarinic acid were found below the reporting limit in all SCG analyzed. Chlorogenic acid, caffeic acid, and syringic acid content were measured at 86.4, 48.1, and 71 mg/100 g, respectively, in espresso coffee grounds, and at 19.4, 20.3, and 17.7 mg/100 g, respectively, in Turkish coffee grounds.

The most well‐known ingredient in coffee and its derivatives is caffeine, or 1,3,7‐trimethylxanthine as it is named technically. Caffeine is a purine alkaloid. Decaffeination, a widely used method in the industry, is how it is removed from coffee beans. Even though coffee by‐products have less caffeine than coffee beans, they nonetheless contain a sizable quantity of caffeine. Espresso coffee grounds showed the highest caffeine content at 181.5 mg/100 g, while filter coffee grounds had the lowest at 6.8 mg/100 g. Caffeine concentrations in wasted coffee ground extracts were reported to range from 0.734 to 41.3 mg/mg in a study. These extracts were made using a variety of extraction techniques, including supercritical fluid CO_2_ extraction and low‐pressure extraction using ultrasound and Soxhlet (Andrade et al. 2012).

Espresso coffee grounds (36.94%) had higher DPPH values than Turkish coffee (26.38%) and filter coffee grounds (13.70%) (*p* < 0.05). Espresso coffee (240.96 mg GAE/100 mL) had the highest (*p* < 0.05) amount of total phenolic compounds (TPC) followed by Turkish coffee (114.95 mg GAE/100 mL) and filter coffee (79.41 mg GAE/100 mL) presented the lowest amount. Pavlović et al. (2013) reported that total phenolic contents of SCG were 17.75 and 21.56 mg GAE/g, respectively.

No significant differences (*p* > 0.05) were observed in total flavonoid content (TFC) values between espresso and Turkish coffee grounds (40.46 and 41.16 mg CE/100 mL, respectively). However, the TFC value of filter coffee grounds was significantly (*p* < 0.05) lower compared to the others. In conclusion, espresso coffee grounds exhibited higher antioxidant activity (DPPH) and total phenolic compound content in spent coffee ground samples, while filter coffee grounds demonstrated the lowest antioxidant activity (DPPH).

Preparation methods significantly influence the antioxidant activity and phenolic compound content of coffee due to variations in extraction parameters such as temperature, pressure, contact time, and coffee‐to‐water ratio. Espresso, prepared under high pressure and temperature within a short duration, typically yields a concentrated extract with high phenolic compound density per milliliter; however, the total antioxidant intake per serving may be lower due to smaller serving sizes (Severini et al. 2018). In contrast, filter coffee involves longer extraction times at lower temperatures, resulting in higher total antioxidant capacity and phenolic content in a standard serving volume (López‐Galilea et al. 2007). Turkish coffee, prepared by boiling finely ground coffee, presents a unique phenolic profile influenced by prolonged heat exposure and sediment retention, which may alter its bioactive compound composition (Vignoli et al. 2011). Collectively, these findings suggest that the choice of brewing technique critically determines the qualitative and quantitative profile of coffee's bioactive compounds, thereby affecting its potential health benefits.

The study evaluated the effects of three grind levels (fine, medium, coarse) and three brewing methods—American (filter), Turkish (boiled), and espresso (pressure)—on coffee's antioxidant activity and total phenolic content. While grind size had a modest influence, the brewing method significantly impacted all measured attributes, with American coffee yielding the highest antioxidant activity and phenolic content per cup compared to espresso and Turkish brews. Caffeine content followed a similar trend, with American coffee showing the greatest total intake, followed by Turkish, then espresso (316, 112, and 64 mg per cup, respectively). Though espresso exhibited the highest concentration of bioactives per milliliter, the larger serving volume of American coffee led to greater overall intake of antioxidant compounds. The authors conclude that brewing method is a key determinant of the health‐related properties of coffee, outweighing the impact of grind level (Derossi et al. 2018).

López‐Galilea et al. (2007) reported that both the brewing method and the roasting type significantly influenced the antioxidant capacity and phenolic profile of coffee beverages. Among the brewing techniques evaluated, espresso demonstrated the highest antioxidant activity, followed by moka, plunger, and filter methods. The study also showed that torrefacto‐roasted coffees had greater antioxidant potential than conventionally roasted ones, likely due to the formation of Maillard reaction products and higher levels of certain phenolics. A strong correlation was observed between total antioxidant capacity and compounds such as caffeine and non‐volatile Maillard derivatives, whereas volatile Maillard products showed little association. These findings suggest that both processing and preparation methods play a critical role in determining the bioactive profile of brewed coffee.

Previous studies using electrochemical methods have demonstrated that both brewing technique and roasting level significantly affect the antioxidant capacity and phenolic content of Turkish and filter coffee. It was reported that light‐roasted filter coffee exhibits higher antioxidant activity, likely due to better preservation of thermolabile phenolic compounds under milder roasting conditions. In contrast, it was suggested that increased roasting intensity leads to the degradation of these bioactives, potentially diminishing the health‐promoting properties of the beverage. These findings, obtained through sensitive voltammetric analyses, highlight the critical role of processing parameters in influencing coffee's functional quality (Yıldırım et al. 2020).

As a result, in the light of the information obtained in this study and literature review, SCG, a globally abundant waste material, exhibits significant potential as a natural resource of phenolic antioxidants due to their substantial content of chlorogenic acid, related compounds, and caffeine (Panusa et al. 2013).

### 3.3 Result of ADMET

The pharmacokinetic properties and safety profiles of the four selected phytochemicals were evaluated through ADMET analysis (Table 4). Molar refractivity values indicated that chlorogenic acid had the highest value (83.50), followed by caffeine (52.04), syringic acid (48.41), and caffeic acid (47.16). Topological polar surface area (TPSA) values were determined as 164.75 Å^2^ for chlorogenic acid, 77.76 Å^2^ for caffeic acid, 75.99 Å^2^ for syringic acid, and 61.82 Å^2^ for caffeine. In terms of consensus Log Po/w, syringic acid (0.99) and caffeic acid (0.93) exhibited a more lipophilic profile, whereas chlorogenic acid (−0.39) had the lowest value.

**TABLE 4:** Acute toxicity profile values of phytochemical components in Turkish, espresso, and filter coffee grounds.

ADMET	Chlorogenic acid	Caffeic acid	Syringic acid	Caffeine
Molar Refractivity	83.50	47.16	48.41	52.04
TPSA	164.75 Å^2^	77.76 Å^2^	75.99 Å^2^	61.82 Å^2^
Consensus Log *P*_o/w_	−0.39	0.93	0.99	0.08
GI absorption	Low	High	High	High
BBB permeant	No	No	No	No
P‐gp substrate	No	No	No	No
CYP1A2 inhibitor	No	No	No	No
CYP2C19 inhibitor	No	No	No	No
CYP2C9 inhibitor	No	No	No	No
CYP2D6 inhibitor	No	No	No	No
CYP3A4 inhibitor	No	No	No	No
Log *K*_p_ (skin permeation)	−8.76 cm/s	−6.58 cm/s	−6.77 cm/s	−7.53 cm/s
Bioavailability Score	0.11	0.58	0.56	0.55
Synthetic accessibility	4.16	1.81	1.70	2.03

Regarding gastrointestinal (GI) absorption, chlorogenic acid exhibited low absorption, while the other three compounds (caffeic acid, syringic acid, and caffeine) demonstrated high absorption potential. Blood–brain barrier (BBB) permeability analysis revealed that none of the compounds could permeate the BBB. Additionally, none of the compounds were identified as P‐glycoprotein (P‐gp) substrates (Figure 3). Inhibitory effects on cytochrome P450 enzymes (CYP1A2, CYP2C19, CYP2C9, CYP2D6, CYP3A4) were also evaluated, and none of the tested compounds exhibited inhibitory activity against these enzymes.

The Log Kp values, representing dermal permeability, were calculated as −8.76, −6.58, −6.77, and −7.53 cm/s for chlorogenic acid, caffeic acid, syringic acid, and caffeine. In terms of bioavailability score, chlorogenic acid had the lowest value (0.11), whereas caffeic acid (0.58), syringic acid (0.56), and caffeine (0.55) exhibited higher bioavailability. Synthetic accessibility was evaluated, with syringic acid showing the lowest complexity (1.70), followed by caffeic acid (1.81), caffeine (2.03), and chlorogenic acid (4.16). These findings provide essential insights into the pharmacokinetic suitability and potential applications of the investigated phytochemicals in drug development processes.

### 3.4 Binding Interactions of Selected Compounds With the 2FLU Target Protein

The binding interactions of the selected phytochemicals with the 2FLU (Kelch‐Neh2 Complex) target protein were evaluated through molecular docking simulations (Table 5). In terms of binding energy, chlorogenic acid exhibited the strongest interaction with a value of −9.3 kcal/mol, followed by caffeic acid (−7.0), syringic acid (−6.6), and caffeine (−6.5 kcal/mol).

**TABLE 5:** Results of binding interactions of the compounds with target 2FLU.

	Binding energy (kcal/mol)	Ligand efficiency	Fit quality (FQ)	Estimated inhibition constant {(Ki) (μM)}	pIC_50_
Chlorogenic acid	−9.3	0.216	0.816	0.152	6.643
Caffeic acid	−7.0	0.333	0.632	7.395	5.000
Syringic acid	−6.6	0.275	0.603	14.527	4.714
Caffeine	−6.5	0.271	0.594	17.198	4.643

Regarding ligand efficiency (LE), caffeic acid showed the highest value (0.333), followed by syringic acid (0.275), caffeine (0.271), and chlorogenic acid (0.216). In terms of fit quality (FQ), chlorogenic acid exhibited the highest fit score (0.816), indicating the best molecular alignment with the binding site, while caffeic acid (0.632), syringic acid (0.603), and caffeine (0.594) demonstrated comparatively lower fit scores.

The estimated inhibition constant (Ki) analysis revealed that chlorogenic acid had the lowest Ki value (0.152 μM), suggesting the highest binding affinity to the target protein. In contrast, caffeic acid (7.395), syringic acid (14.527), and caffeine (17.198 μM) exhibited higher Ki values, indicating weaker binding affinities.

In terms of pIC50 values, chlorogenic acid demonstrated the highest inhibitory potential (6.643), followed by caffeic acid (5.000), syringic acid (4.714), and caffeine (4.643). These findings suggest that chlorogenic acid exhibits the strongest interaction with the 2FLU target protein and may serve as a promising inhibitor candidate.

**FIGURE 1:**
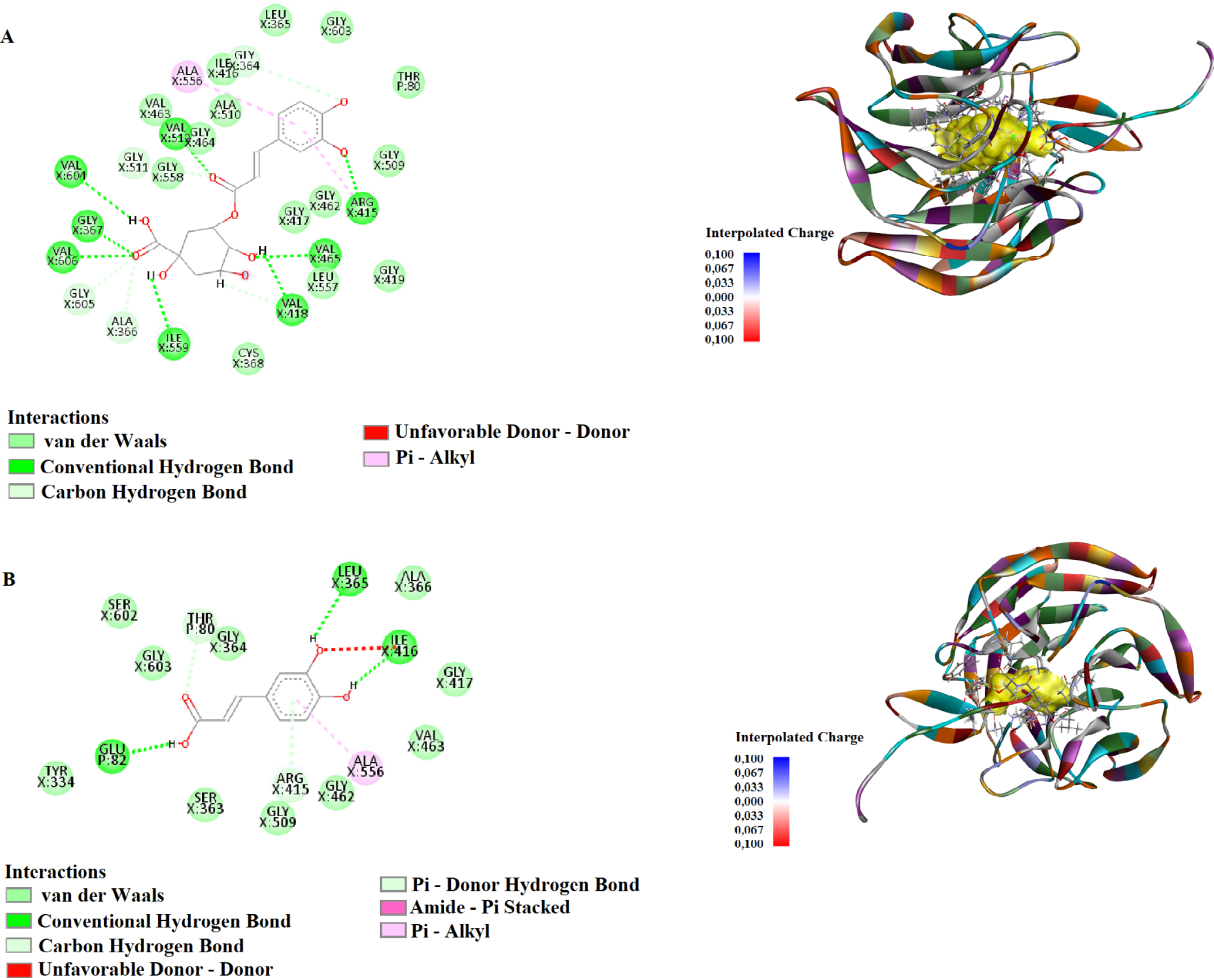
Molecular docking analysis of 2D and 3D interactions of (A) Chlorogenic acid (B) Caffeic acid with their respective target 2FLU.

Molecular docking simulations were conducted to examine the binding interactions of the selected compounds with the 2FLU target protein (Table 5). Chlorogenic acid demonstrated strong interactions within the binding pocket, forming conventional hydrogen bonds with Gly367, Arg415, Val465, Val512, Val606, Val418, and Ile559 at distances ranging from 1.58 to 2.53 Å. The strongest hydrogen bond was observed with Gly367 at 1.58 Å. Additionally, Pi‐alkyl interactions were detected with Arg415 and Ala556 at 4.76 and 5.19 Å, respectively (Figure 1A, Table 6).

**TABLE 6:** Docking of predicted interactions of docked conformations of compounds against 2FLU.

Ligand	Amino acids	Interacting	Distance
Chlorogenic acid	X:GLY367:HN—:[001:O8	Conventional Hydrogen Bond	1.58
	X:ARG415:HE—:[001:O5	Conventional Hydrogen Bond	2.14
	X:VAL465:HN—:[001:O2	Conventional Hydrogen Bond	2.53
	X:VAL512:HN—:[001:O6	Conventional Hydrogen Bond	2.09
	X:VAL606:HN—:[001:O8	Conventional Hydrogen Bond	1.91
	:[001:H5—X:VAL418:O	Conventional Hydrogen Bond	1.76
	:[001:H17—X:VAL604:O	Conventional Hydrogen Bond	2.48
	:[001:H18—X:ILE559:O	Conventional Hydrogen Bond	2.44
	X:GLY364:HA1—:[001:O4	Carbon Hydrogen Bond	2.69
	X:ALA366:HA—:[001:O8	Carbon Hydrogen Bond	2.54
	X:GLY511: HA1—:[001:O6	Carbon Hydrogen Bond	2.63
	X:GLY605: HA1—:[001:O8	Carbon Hydrogen Bond	2.41
	:[001:H4—X:VAL418:O	Carbon Hydrogen Bond	2.56
	:[001—X:ARG415	Pi‐Alkyl	4.76
	:[001—X:ALA556	Pi‐Alkyl	5.19
Caffeic acid	:[001:H1—P:GLU82:OE2	Conventional Hydrogen Bond	1.77
	:[001:H6—X:ILE416:O	Conventional Hydrogen Bond	1.68
	:[001:H7—X:LEU365:O	Conventional Hydrogen Bond	2.13
	P:THR80:HB—:[001:O1	Carbon Hydrogen Bond	2.38
	X:ARG415:HE—:[001	Pi‐Donor Hydrogen Bond	3.11
	X:ARG415:C,O;ILE416:N—:[001	Amide‐Pi Stacked	4.55
	:[001—X:ARG415	Pi‐Alkyl	4.06
	:[001—X:ALA556	Pi‐Alkyl	5.25
Syringic acid	X:GLY367:HN—:[001:O4	Conventional Hydrogen Bond	1.70
	X:VAL418:HN—:[001:O1	Conventional Hydrogen Bond	2.24
	:[001:H1—X:ILE416:O	Conventional Hydrogen Bond	1.65
	:[001:H6—X:VAL606:O	Conventional Hydrogen Bond	1.92
	X: GLY417:HA1—:[001:O1	Carbon Hydrogen Bond	2.39
	X:GLY558:HA1—:[001:O5	Carbon Hydrogen Bond	2.91
	:[001:H3—X:LEU365:O	Carbon Hydrogen Bond	2.47
	:[001:H8—X:VAL418:O	Carbon Hydrogen Bond	3.05
	:[001:H8—X:VAL465:O	Carbon Hydrogen Bond	2.56
	:[001:C9—X:VAL465	Alkyl	4.43
	:[001—X:ALA366	Pi‐Alkyl	3.61
Caffeine	X:ARG415:HH21—:[001:N3	Conventional Hydrogen Bond	2.01
	X:GLY364:HA1—:[001:O2	Carbon Hydrogen Bond	2.74
	X:GLY509:HA2—:[001:N3	Carbon Hydrogen Bond	2.84
	X:GLY603:HA2—:[001:O2	Carbon Hydrogen Bond	2.47
	:[001:H3—:[001:O2	Carbon Hydrogen Bond	2.49
	:[001:H6—X:ALA510:O	Carbon Hydrogen Bond	2.39
	:[001:H7—X:GLY462:O	Carbon Hydrogen Bond	3.07
	:[001:H8—X:VAL604:O	Carbon Hydrogen Bond	2.98
	:[001:H10—X:LEU365:O	Carbon Hydrogen Bond	2.06
	X:ALA556—:[001:C6	Alkyl	3.81
	:[001:C6—X:ARG415	Alkyl	4.57
	:[001—X:ARG415	Pi‐Alkyl	4.27
	:[001—X:ALA556	Pi‐Alkyl	5.15
	:[001—X:ALA556	Pi‐Alkyl	3.80

Caffeic acid exhibited interactions with Glu82, Ile416, Leu365, Thr80, Arg415, and Ala556 residues. Conventional hydrogen bonds were identified at distances between 1.68 and 2.13 Å, with the strongest interaction observed with Ile416 at 1.68 Å. Moreover, Pi‐donor hydrogen bonds and amide‐Pi stacking interactions were present, contributing to ligand stability. Pi‐alkyl interactions were formed with Arg415 and Ala556 at 4.06 and 5.25 Å, respectively (Figure 1B).

**FIGURE 2:**
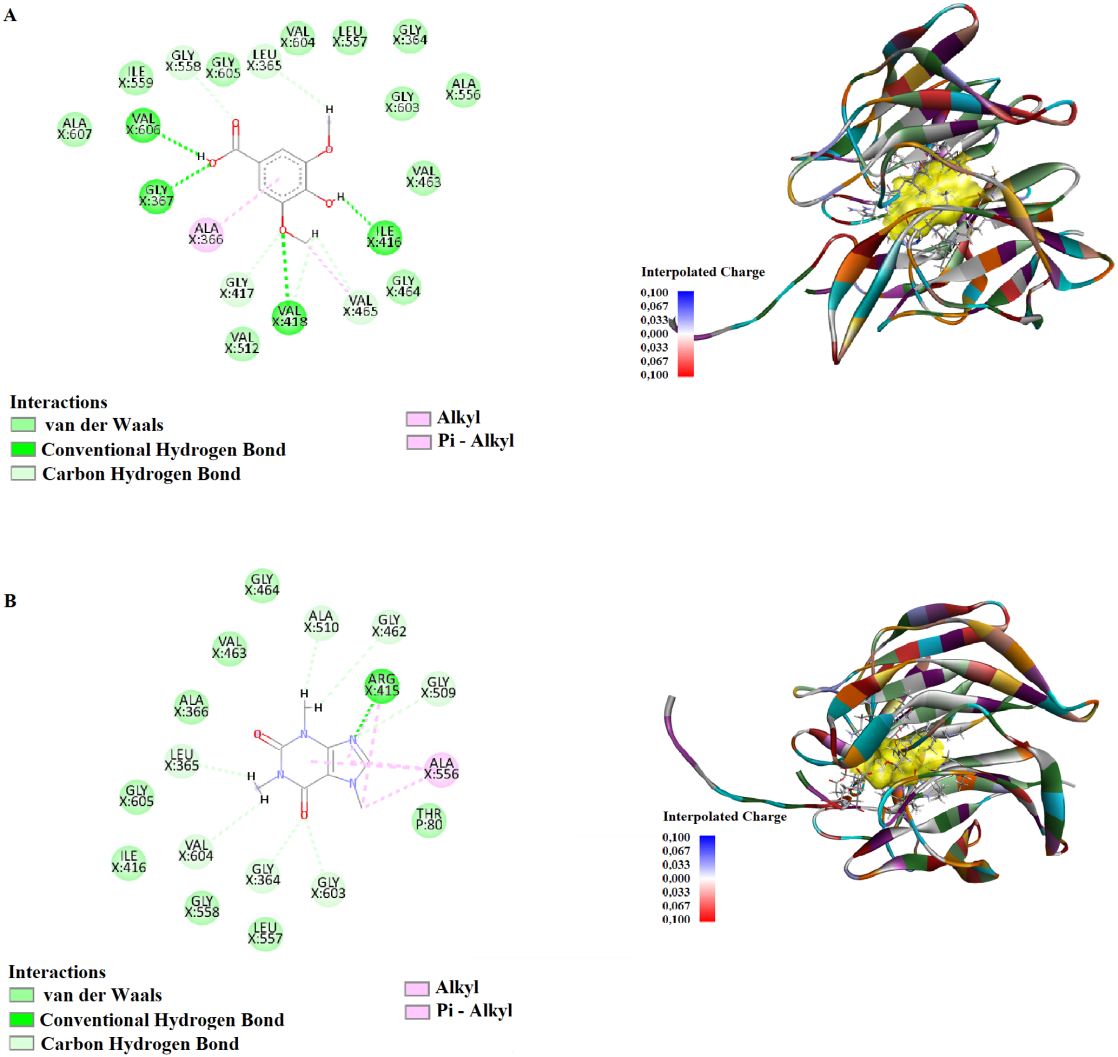
Molecular docking analysis of 2D and 3D interactions of (A) Syringic acid (B) Caffeine with their respective target 2FLU.

Syringic acid interacted with Gly367, Val418, Ile416, Val606, Gly417, Gly558, Leu365, and Val465 amino acids. The conventional hydrogen bonds were established at distances between 1.65 and 2.24 Å, with the strongest interaction observed at 1.65 Å with Ile416. Additionally, carbon‐hydrogen bonds ranged from 2.39 to 3.05 Å, while alkyl and Pi‐alkyl interactions were observed with Ala366 and Val465 at 3.61 and 4.43 Å, respectively (Figure 2A).

Caffeine formed interactions with Arg415, Gly364, Gly509, Gly603, Ala510, Gly462, Val604, Leu365, and Ala556 residues. The strongest conventional hydrogen bond was observed with Arg415 at 2.01 Å, while carbon‐hydrogen bonds were found within 2.06 to 3.07 Å. Pi‐alkyl and alkyl interactions were also detected with Arg415 and Ala556, ranging from 3.80 to 5.15 Å (Figure 2B).

These findings highlight distinct binding patterns among the investigated compounds, with chlorogenic acid and caffeic acid displaying stronger interactions with the 2FLU target protein, suggesting their potential as promising inhibitors.

### 3.5 Physical Analyses of Samples

The water activity, cooking loss, and diameter reduction values of the samples are shown in Table 7.

**TABLE 7:** Cooking loss, diameter reduction, and water activity parameters of beef patties.

Analysis	Treatments	Storage period (day)			Statistical Significance
		1	3	7	Interaction of treatments and storage period
Cooking loss (%)	C	17.19 ± 0.31^BCD^	9.95 ± 0.61^G^	10.81 ± 0.15^FG^	
	T1	16.25 ± 1.20^CDE^	18.50 ± 1.00^ABCD^	11.86 ± 0.39^EFG^	*
	T2	23.13 ± 0.32^A^	20.79 ± 0.47^ABC^	14.79 ± 1.43^DEF^	
	T3	17.42 ± 1.15^BCD^	19.84 ± 0.89^ABC^	21.12 ± 1.17^AB^	
Diameter reduction (%)	C	33.00 ± 0.22	15.63 ± 1.80	16.18 ± 1.15	
	T1	35.68 ± 4.21	19.93 ± 0.88	18.88 ± 0.00	ns
	T2	36.53 ± 0.29	21.45 ± 1.90	19.00 ± 2.14	
	T3	31.45 ± 2.72	20.68 ± 1.94	17.96 ± 2.40	
Water activity	C	0.980 ± 0.00^DEF^	0.979 ± 0.00^EF^	0.978 ± 0.00^F^	
	T1	0.988 ± 0.00^A^	0.987 ± 0.00^AB^	0.984 ± 0.00^BCD^	*
	T2	0.986 ± 0.00^ABC^	0.988 ± 0.00^A^	0.983 ± 0.00^CD^	
	T3	0.987 ± 0.00^AB^	0.989 ± 0.00^A^	0.982 ± 0.00^DE^	

*Note:* Mean ± standard error. ^A–G^Different letters indicate statistically significant differences among means within beef patty samples across storage periods.

Abbreviations: C, Control; ns, not significant; T1, Turkish coffee ground added beef patty; T2, Espresso coffee ground added beef patty; T3, Filter coffee ground added beef patty.

* *p* < 0.05.

The water activity values of the samples were determined between 0.989 and 0.978, and the interaction of the use of SCG in the formulation and storage process was found to be significant (*p* < 0.05). The use of SCG in the formulation resulted in higher water activity compared to the control; the lowest water activity value was measured on the 7th day of the control group samples. Beef patty samples had similar aw values with the literature (Martuscelli et al. 2021).

In meat and meat products, “cooking loss” refers to the weight loss that occurs in meat as a result of cooking or processing. This loss usually occurs as water loss and is calculated as the difference between the initial weight of the meat and the weight remaining after cooking (Purslow et al. 2016).

The interaction of storage period and SCG treatment was found to be significant (*p* < 0.05) and the highest cooking loss was determined in T2 samples on day 1, and the lowest cooking loss was determined in control group samples on day 3.

The use of SCG in beef patty samples had no effect (*p* > 0.05) on diameter reduction compared to the control group.

Some research in the literature (Choi et al. 2010, 2016; Turhan et al. 2005) suggests that incorporating dietary fiber into meat and meat products can help reduce cooking losses and diameter reduction values. However, these studies have primarily focused on reduced‐fat meat products. Cooking loss is associated with the ability of meat proteins, fat, and moisture to bind together. In our study, the different outcomes are likely due to the fact that the fat content in the formulation remained unchanged and coffee grounds were used as the dietary fiber source.

**FIGURE 3:**
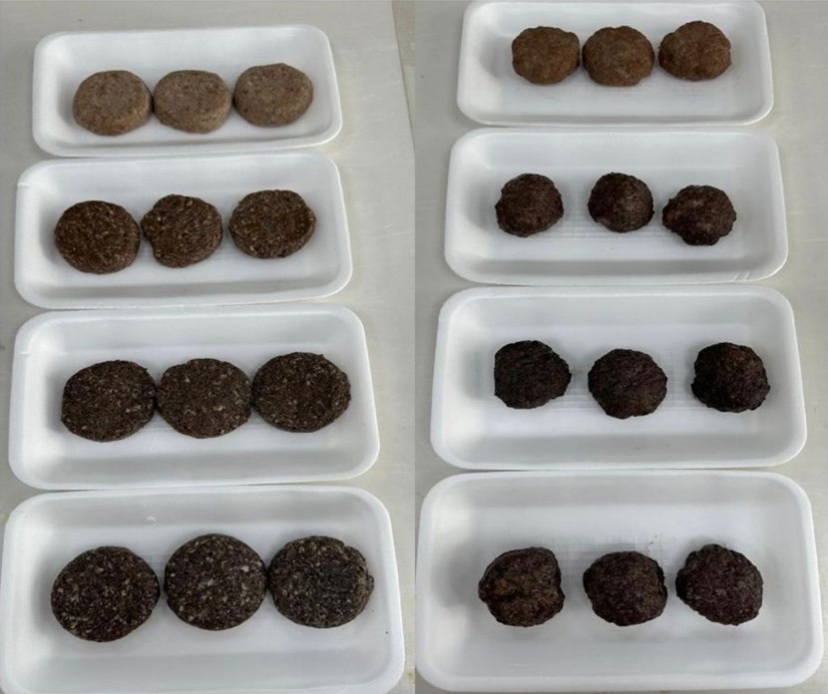
Photographs of raw and cooked beef patties with different spent coffee grounds. *From top to bottom: C, Control; T1, Turkish coffee ground added beef patty; T2, Espresso coffee ground added beef patty; T3, Filter coffee ground added beef patty. From left to right: Raw and cooked beef patties.

According to Table 8, where pH values and color parameters of raw and cooked beef patties are shown, the interaction of storage period and SCG treatment was found to be significant (*p* < 0.05) in terms of L*, a*, and b* values of beef patty samples, both raw and cooked ones. The highest L*, a*, and b* values were observed in the control group samples. Both raw and cooked control group samples exhibited the highest L* value on all analysis days and the highest a* value on day 1. The highest b* values were observed on day 7 for cooked samples and on all analysis days for raw samples. The treatment groups have lower L* values arising from the dark color typical of coffee grounds used instead of breadcrumbs, and it is an expected outcome (Figure 3).

**TABLE 8:** pH values and color properties of raw and cooked beef patties.

Parameters	Treatments	Storage period (day)						Statistical Significance	
		1		3		7		Interaction of treatments and storage period for raw samples	Interaction of treatments and storage period for cooked samples
		Raw	Cooked	Raw	Cooked	Raw	Cooked		
L*	C	57.1 ± 0.92^A^	46.0 ± 0.68^A^	56.5 ± 0.11^A^	46.1 ± 0.61^A^	56.1 ± 0.72^A^	45.4 ± 0.47^A^		
	T1	45.5 ± 0.28^B^	41.3 ± 0.37^bc^	40.7 ± 0.16^CD^	39.1 ± 0.02^CD^	36.8 ± 0.14^F^	41.2 ± 0.76^bc^	*	*
	T2	41.6 ± 0.04^CD^	38.0 ± 0.23^DE^	38.0 ± 0.03^EF^	38.6 ± 0.45^CD^	35.8 ± 0.13^F^	38.8 ± 0.26^CD^		
	T3	41.9 ± 0.47^C^	39.7 ± 0.27^BCD^	39.4 ± 0.17^DE^	35.6 ± 0.54^E^	37.6 ± 0.71^EF^	42.3 ± 0.82^B^		
a*	C	13.0 ± 0.04^A^	9.4 ± 0.03^A^	9.5 ± 0.16^B^	8.3 ± 0.01^B^	5.5 ± 0.01^H^	6.3 ± 0.13^CD^		
	T1	7.8 ± 0.00^D^	8.4 ± 0.05^B^	8.8 ± 0.07^C^	6.9 ± 0.13^C^	6.8 ± 0.01^E^	4.2 ± 0.14^F^	*	*
	T2	5.9 ± 0.01^GH^	5.2 ± 0.06^E^	6.1 ± 0.01^G^	4.8 ± 0.02^E^	6.7 ± 0.17^EF^	4.0 ± 0.01^F^		
	T3	5.9 ± 0.11^GH^	5.9 ± 0.15^D^	6.2 ± 0.01^FG^	3.9 ± 0.16^F^	6.8 ± 0.21^E^	2.1 ± 0.04^G^		
b*	C	17.7 ± 0.13^A^	13.8 ± 0.69^B^	18.4 ± 0.11^A^	14.4 ± 0.32^AB^	18.2 ± 0.33^A^	15.8 ± 0.74^A^		
	T1	13.4 ± 0.27^C^	13.1 ± 0.04^B^	14.5 ± 0.06^B^	9.0 ± 0.04^C^	11.6 ± 0.09^D^	7.4 ± 0.11^CD^	*	*
	T2	10.0 ± 0.04^E^	8.8 ± 0.09^C^	13.6 ± 0.07^bc^	7.6 ± 0.06^CD^	13.5 ± 0.12^bc^	6.0 ± 0.08^D^		
	T3	9.7 ± 0.05^E^	8.5 ± 0.18^C^	11.4 ± 0.16^D^	8.5 ± 0.54^C^	9.0 ± 0.36^E^	5.8 ± 0.15^D^		
pH	C	5.4 ± 0.02^B^	5.6 ± 0.01^D^	5.1 ± 0.01^C^	5.1 ± 0.01^E^	4.8 ± 0.01^C^	4.9 ± 0.03^F^		
	T1	5.5 ± 0.03^AB^	5.7 ± 0.00^C^	5.5 ± 0.03^AB^	5.7 ± 0.01^C^	5.8 ± 0.17^A^	6.1 ± 0.01^A^	*	*
	T2	5.5 ± 0.00^AB^	5.7 ± 0.01^C^	5.5 ± 0.01^AB^	5.7 ± 0.01^C^	5.7 ± 0.02^AB^	6.0 ± 0.00^B^		
	T3	5.5 ± 0.01^AB^	5.7 ± 0.01^C^	5.6 ± 0.01^AB^	5.7 ± 0.01^C^	5.8 ± 0.02^A^	6.1 ± 0.03^A^		

*Note:* Mean ± standard error. ^A–H^Different letters indicate statistically significant differences among means within beef patty samples with the same title (raw or cooked) across the storage period.

Abbreviations: C, Control; ns, not significant; T1, Turkish coffee ground added beef patty; T2, Espresso coffee ground added beef patty; T3, Filter coffee ground added beef patty.

* *p* < 0.05.

Martuscelli et al. (2021) in their study on chicken meat burgers with the addition of coffee silver skin (another by‐product of coffee), the L*, a*, and b* values of samples containing 1.5% and 3% coffee silver skin were found to be 32.8% and 29.4%, 3.8% and 4.9%, 13.7% and 14.6%, respectively, and 47.4% and 43.5%, 2.3% and 2.8%, 10.9% and 12.1% for cooked samples. The beef patty samples containing SCG that we produced in this study are actually darker in color than the control. Unfortunately, there are no similar studies to compare these data.

The interaction of storage period and SCG treatment was found to be significant (*p* < 0.05) in terms of the pH values of samples, and the highest pH values were measured in T1 and T3 samples on day 7 for both raw and cooked samples, while the lowest pH values were determined in control group samples on day 7 for cooked ones and on days 3 and 7 for raw ones.

From a food safety perspective, the pH of meat products should typically not drop below 5.0. (Sofos 2014). Therefore, the effect of SCG in the formulation, resulting in an increase in pH values (Table 8), can be considered beneficial.

Interestingly, although SCG are generally characterized by a slightly acidic pH (typically ranging between 5.0 and 5.5) (Mussatto et al. 2011), the incorporation of SCG into the meat matrix resulted in higher pH values compared to the control samples. This observation can be explained by several interrelated factors. First, the presence of melanoidins and dietary fibers formed during the roasting process may confer buffering capacity, limiting the expected decrease in pH due to their interaction with acidic components in the meat matrix (del Castillo et al. 2002; Moreira et al. 2012). Second, dark roasting conditions can lead to the thermal degradation of acidic compounds such as chlorogenic acids, thus reducing the net acidity of SCG (Vignoli et al. 2011). Furthermore, SCG contains minerals such as potassium, calcium, and magnesium, which may contribute to an overall alkalizing effect (Ballesteros et al. 2014). In addition, the antioxidant activity of SCG might have inhibited microbial growth and subsequent acid production during storage, thereby maintaining a higher pH. These results align with previous studies reporting that certain plant‐based additives can exert similar buffering and stabilizing effects on pH in meat systems (Zhao et al. 2019).

### 3.6 TBARS Numbers of Samples

As shown in Table 9, the interaction of storage period and SCG treatment was found to be significant (*p* < 0.05) in terms of the TBARS numbers of samples. The highest TBARS number was measured in the control group on day 7, as expected, and Turkish coffee ground had the highest effect on TBARS numbers during the storage period, while the lowest TBARS number was determined on the 7th day of storage.

**TABLE 9:** The TBARS number (mg MA/kg sample) of beef patties.

Analysis	Treatments	Storage period (day)			Statistical Significance
		1	3	7	Interaction of treatments and storage period
TBARS number (mg MA/kg sample)	C	0.92 ± 0.00^C^	1.07 ± 0.00^B^	2.17 ± 0.01^A^	
	T1	0.64 ± 0.01^DE^	0.57 ± 0.01^E^	0.55 ± 0.05^E^	*
	T2	0.69 ± 0.00^DE^	0.71 ± 0.04^D^	0.68 ± 0.05^DE^	
	T3	0.57 ± 0.02^DE^	0.63 ± 0.03^DE^	0.56 ± 0.03^E^	

*Note:* Mean ± standard error. ^A–E^Different letters indicate statistically significant differences among means within beef patty samples across the storage period.

Abbreviations: C, Control; ns, not significant; T1, Turkish coffee ground added beef patty; T2, Espresso coffee ground added beef patty; T3, Filter coffee ground added beef patty.

* *p* < 0.05.

It is a very important result that the treatment groups shown as T1, T2, and T3 had significantly lower (*p* < 0.05) TBARS numbers than the control group throughout the storage period. All beef patties with SCG had TBARS numbers that were below the permissible sensory threshold limit for having a rancid flavor (1 mg/kg) while this limit was exceeded limit in the control group from the 3rd day. As a result, it is thought that the use of these SCG can inhibit oxidative reactions during the storage period and thus increase the quality of beef patties. This antioxidant activity, which is effective in reducing the TBARS numbers of the samples, may be related to the phenolic compounds abundant in SCG. In the literature, it is reported that silver skin, SCG, and cherry husk are rich in phenolic compounds, and chlorogenic acid is the most important phenolic component (Esquivel and Jimenez 2012; Murthy and Naidu 2012).

When de Farias Marques et al. (2022) examined chicken patties supplemented with organic coffee husk extract, another coffee byproduct, they discovered results comparable to those presented in this study. When the storage period came to an end, the coffee husk extract‐added samples' TBARS levels were noticeably lower than those of the control group (no antioxidant).

Coffee husks have been found to reduce lipid oxidation in fresh sausages, especially in MAP packaging, and can be evaluated as antioxidants in fresh meat products. Another study was carried out to assess the antioxidant effects of coffee husks on the physicochemical properties and sensory liking of fresh sausages with different packaging methods, aerobic (AEP) or modified atmosphere packaging (MAP) (Araya‐Morice et al. 2023). Similar results supporting our study were obtained in a study using extract of the residue of ground coffee obtained after the brewing process (Kim et al. 2016). These researchers reported that the ethanol extracts of SCG or coffee residues, by another name, have a strong antioxidant activity and have the potential to be used as a natural antioxidant in meat instead of synthetic antioxidants.

### 3.7 Dietary Fiber Content of Samples

**FIGURE 4:**
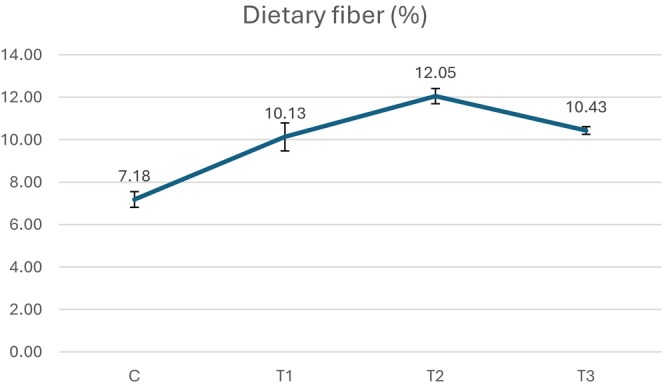
Dietary fiber contents of beef patties. C, Control; T1, Turkish coffee ground added beef patty; T2, Espresso coffee ground added beef patty; T3, Filter coffee ground added beef patty.

Figure 4 shows the dietary fiber content of the samples. The dietary fiber in the control group samples without SCG was significantly lower (7.18%) compared to the treatment groups. The amounts of dietary fiber in the treatment groups were 10.13%, 12.05%, and 10.43% for T1, T2, and T3, respectively, and there was no significant (*p* > 0.05) difference between them.

SCG contain between 45 and 51 g of total dietary fiber per 100 g, consisting of 35 to 48 g of insoluble fibers and 2–8 g of soluble fibers (Vilela et al. 2016). This total dietary fiber content places SCG in a similar range as fiber‐rich powders derived from fruit peels like orange, prickly pear, and feijoa (de Almeida et al. 2020; Tejada‐Ortigoza et al. 2017). However, SCG has higher total dietary fiber content compared to some commonly used fiber sources like rice and wheat bran (Elleuch et al. 2011) as well as certain fruit peel powders including pequi and mango (Leão et al. 2017; Tejada‐Ortigoza et al. 2017).

The beneficial health effects of dietary fibers in combating lifestyle diseases such as cardiovascular diseases, cancer, diabetes, and obesity are well documented. Adding fibers to meat products significantly impacts cooking yield, water holding capacity (WHC), juiciness, color, and texture, and can serve as a substitute for fat. Meat scientists have explored various sources of dietary fiber to create nutritious, healthier, and functional products with favorable taste and proven effectiveness. It is crucial in our era to innovate meat products enriched with dietary fiber to tackle the rising prevalence of lifestyle‐related diseases. Future research could focus on identifying new sources of dietary fibers rich in bioactive compounds to enhance the development of functional meat products.

SCG can be used directly as a source of natural dietary fiber. However, it can also be extracted from the raw material using different processes such as ohmic heating, alkaline hydrogen peroxide treatment, and autohydrolysis. The antioxidant activity of coffee fibers may persist after treatment with these processes. The metabolites formed by intestinal fermentation of SCG show a strong anti‐inflammatory effect by inhibiting nitric oxide production and the production of some cytokines that are inflammatory mediators. In addition, coffee dietary fibers have been found to stimulate the release of satiety hormone, serotonin, and glucagon‐like peptide‐1 (GLP‐1). In addition, it has been reported that antioxidant polysaccharides extracted from SCG via autohydrolysis can be used in the encapsulation of other bioactive food components such as prebiotics (Ballesteros et al. 2017).

SCG have been used as a source of dietary fiber in the production of biscuits (Campos‐Vega et al. 2020), muffins (Severini et al. 2020), beverages (Sampaio et al. 2013) and cookies (Desai et al. 2020), but no studies on their use in meat and meat products as a dietary fiber source were found in the literature. Therefore, the results obtained in this study are important because they offer an alternative to natural sources used in the enrichment of meat products in terms of dietary fiber, and their evaluation is important in many aspects, especially for economic and environmental reasons.

### 3.8 Textural Properties of Samples

The overall acceptance of meat products heavily relies on their sensory attributes, color characteristics, and especially texture properties. Incorporating fiber into meat products significantly modifies their texture, color, tenderness, flavor, and juiciness. Studies consistently show that regardless of the intended purpose of fiber addition, it enhances the functional properties and health benefits of meat products (Younis et al. 2022). These variations are primarily influenced by the type of fiber (soluble/insoluble), its source (fruits, vegetables, cereals, legumes, etc.), and the amount added.

Table 10 presents the effect of SCG application and a 7‐day storage period on the texture properties of beef patty samples. Hardness, a parameter used in the determination of texture properties in meat products, is defined as the force required to compress a food between the molars or the force required to achieve a certain deformation (Novaković and Tomašević 2017).

**TABLE 10:** Textural properties of beef patties.

Parameters	Treatments	Storage period (day)			Statistical Significance
		1	3	7	Interaction of treatments and storage period
Hardness (N)	C	92.69 ± 1.42^G^	104.32 ± 1.19^F^	153.57 ± 0.68^A^	
	T1	94.68 ± 0.62^G^	93.08 ± 0.10^G^	91.16 ± 0.62^G^	*
	T2	140.74 ± 0.16^bc^	124.09 ± 1.46^D^	133.45 ± 1.90^C^	
	T3	113.41 ± 3.03^E^	116.69 ± 2.40^DE^	142.60 ± 2.18^B^	
Springiness	C	0.77 ± 0.02	0.73 ± 0.01	0.69 ± 0.01	
	T1	0.72 ± 0.00	0.67 ± 0.02	0.74 ± 0.00	ns
	T2	0.75 ± 0.01	0.71 ± 0.02	0.77 ± 0.02	
	T3	0.69 ± 0.00	0.71 ± 0.07	0.73 ± 0.00	
Cohesiveness	C	0.58 ± 0.04^AB^	0.48 ± 0.00^DE^	0.46 ± 0.00^E^	
	T1	0.52 ± 0.00^BCDE^	0.58 ± 0.00^AB^	0.56 ± 0.00^ABC^	*
	T2	0.56 ± 0.01^ABC^	0.51 ± 0.01^CDE^	0.54 ± 0.01^BCD^	
	T3	0.52 ± 0.01^BCDE^	0.52 ± 0.00^BCDE^	0.62 ± 0.00^A^	
Gumminess (N)	C	53.67 ± 4.24^DEFG^	49.94 ± 0.54^FG^	70.96 ± 0.37^bc^	
	T1	49.17 ± 0.26^G^	53.55 ± 0.93^DEFG^	51.16 ± 0.02^EFG^	*
	T2	78.43 ± 1.39^B^	62.66 ± 2.43^CD^	72.00 ± 1.90^bc^	
	T3	58.95 ± 0.95^DEF^	60.44 ± 0.69^DE^	88.42 ± 1.68^A^	
Chewiness (N)	C	41.51 ± 4.25^DE^	36.48 ± 0.07^DE^	48.67 ± 1.36^BCD^	
	T1	35.24 ± 0.06^E^	35.90 ± 0.64^E^	37.72 ± 0.06^DE^	*
	T2	58.41 ± 0.49^AB^	44.40 ± 2.96^CDE^	55.60 ± 2.98^ABC^	
	T3	40.61 ± 0.68^DE^	42.87 ± 4.10^DE^	65.09 ± 1.68^A^	
Resilience	C	0.25 ± 0.02^AB^	0.18 ± 0.00^DE^	0.16 ± 0.00^E^	
	T1	0.21 ± 0.00^BCD^	0.25 ± 0.00^ABC^	0.25 ± 0.00^ABC^	*
	T2	0.22 ± 0.01^BCD^	0.19 ± 0.00^DE^	0.21 ± 0.00^BCD^	
	T3	0.20 ± 0.01^CDE^	0.21 ± 0.01^BCD^	0.26 ± 0.00^A^	

*Note:* Mean ± standard error. ^A–G^Different letters indicate statistically significant differences among means within beef patty samples across storage periods.

Abbreviations: C, Control; ns, not significant; T1, Turkish coffee ground added beef patty; T2, Espresso coffee ground added beef patty; T3, Filter coffee ground added beef patty.

* *p* < 0.05.

On the 1st and 3rd day of the study, the use of espresso and filter coffee ground in beef patty production resulted in harder patties compared to the control samples. However, on the last day of the study, SCG treatment decreased the hardness of the samples, and the highest hardness was found in the control group (*p* < 0.05). Beef patties formulated with Turkish coffee ground were found to have lower hardness values on all analysis days. This may be explained by the softer texture of Turkish coffee ground due to its smaller particle content compared to espresso or filter coffee ground.

There is no significant (*p* > 0.05) effect of storage duration and SCG treatments on the springiness parameters, defined as the rate at which a deformed material returns to its original state or undeformed after the deforming force is removed, of the samples.

In meat products, cohesiveness is a parameter that expresses the interconnectedness of the components in the product and the ability of these components to hold together. This property has an important influence on the texture and structural integrity of the product. A high cohesiveness value in meat products ensures that the product has a homogeneous structure and can be presented to the consumer without falling apart or disintegrating. This is important for ease of use and consumer satisfaction (Novaković and Tomašević 2017).

Cohesiveness helps meat products to have a consistent mouthfeel and to exhibit the desired structural properties during chewing. This parameter is an important criterion used to determine the interaction of fibers and other components in the product and the quality of the product. Meat products with a high cohesiveness value generally offer a more satisfying consumption experience and increase the marketability of the product (García‐Segovia et al. 2014). As shown in Table 10, the interaction of storage period and SCG treatment was found to be significant (*p* < 0.05) in terms of the cohesiveness of samples. The highest cohesiveness value was measured in the T3 group on day 7 during the storage period, and the lowest one was determined in the control group on the 7th day of storage.

When the samples are compared in terms of gumminess, a parameter indicating the sticky or elastic sensation experienced during chewing of meat products, the highest gumminess value was measured in the T3 group on day 7 and the lowest one was determined in the T1 group on the 1st day of storage. Excessive gumminess can be an undesirable characteristic for consumers, especially in processed meat products. Maintaining a natural texture and flavor perception is crucial for meat products, emphasizing the importance of balanced gumminess values influenced by factors such as ingredients, processing methods, and storage conditions.

In evaluating the chewiness parameter of beef patty samples, the interaction of storage period and SCG treatment was found to be significant (*p* < 0.05). The highest chewiness value was measured in the T3 group on day 7, and the lowest one was determined in the T1 group on the 1st and 3rd days of storage.

According to Table 10, the interaction of storage period and SCG treatment was found to be significant (*p* < 0.05) in terms of the resilience parameter. The highest resilience value was measured in the T3 group, and the lowest one was determined in the control group on day 7.

de Farias Marques et al. (2022) added organic coffee husk extract to the chicken burger formulation in an amount of 100 and 200 ppm CAE equivalents, and the toughness values of the chicken burgers obtained were 16.54 ± 0.01 and 16.53 ± 0.44, the elasticity values were 0.93 ± 0.001 and 0.92 ± 0.001, the cohesiveness values were 0.77 ± 0.01 and 0.78 ± 0.001, the gumminess values were 12.45 ± 0.84 and 13.96 ± 0.58, the chewiness values were 10.69 ± 1.05 and 11.59 ± 0.48, and the resilience values were 0.39 ± 0.04 and 0.40 ± 0.01 reported. The differences in these study results compared to our data can be attributed to the use of a different coffee by‐product (coffee husk) and the utilization of its extract rather than the residue itself in the formulation.

Although the use of SCG to enrich meat and meat products in terms of dietary fiber is not found in the literature, there are studies using waste products rich in dietary fiber such as psyllium husks (Mehta et al. 2013), citrus fiber (Petridis et al. 2014) and rice bran (Petridis et al. 2014) for this purpose.

In a study evaluating the effect of the addition of dried carrot pulp and wheat bran at 3%, 6%, and 9% levels on texture properties such as stickiness, hardness, gumminess, chewiness, and springiness, it was reported that wheat bran or dried carrot pulp caused a hard texture in chicken sausages and the hardness value of the samples increased depending on the wheat bran and dried carrot pulp content, while the springiness and stickiness gradually decreased with increasing levels of dried carrot pulp. The increase was explained by the presence of a dense network structure within the meat matrix caused by the higher content of insoluble fiber in chicken sausage, which typically has a strong capacity for binding water (Yadav et al. 2018). Nitin Mehta et al. (2013) noted that as the amount of psyllium husk added to chicken patties increased, all texture parameters decreased. Specifically, the tenderness of the patties decreased with higher husk content, which they attributed to the softening effect caused by the inclusion of soluble dietary fiber.

### 3.9 Sensory Traits of Samples

**FIGURE 5:**
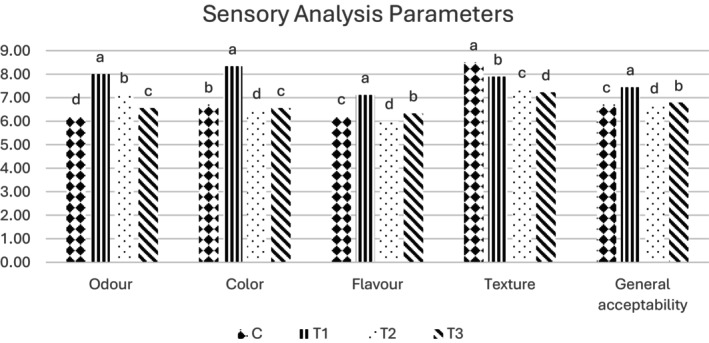
Sensory scores of the beef patties (C, Control; T1, Turkish coffee ground added beef patty; T2, Espresso coffee ground added beef patty; T3, Filter coffee ground added beef patty). Bar charts with different letters indicate significant differences between the treatments (a–d) in each sensory parameter (*p* < 0.05).

The sensory analysis conducted to determine the consumer acceptability and edibility of the beef patties produced in the study revealed that the use of SCG in the formulation had a positive effect (*p* < 0.05) on the odor properties of the samples. Specifically, the use of Turkish coffee grounds, among other types of SCG, improved all sensory parameters except for texture characteristics (*p* < 0.05). There were adverse effects of SCG on the texture (*p* < 0.05). This is due to the fibrous and porous structure of the SCG, which prevents homogeneous binding of the beef patties, and its low water holding capacity compared to meat. However, considering overall acceptability, samples added Turkish and filter coffee grounds received higher scores compared to the control group (Figure 5). Contrary to our results, a study using coffee silver skin (another by‐product of coffee) in chicken patties (Özhamamcı 2024) and studies on various meat products formulated with different dietary fibers (Barros et al. 2018; Choi et al. 2016; Ferjančič et al. 2021; Kurt and Kılınççeker 2018) have reported lower average values for sensory parameters.

## 4 Conclusion

SCG have versatile functional properties like prebiotic content and highly nutritional composition in terms of vitamins, minerals, carbohydrates, proteins, and lipids. Additionally, its high dietary fiber content makes it attractive for use in foods, particularly since meat products typically lack sufficient dietary fiber. Thanks to its bioactive components, when used in meat products, it protects the product against oxidative stress as well as providing a functional product. The results showed that SCG of Turkish, espresso, and filter coffee incorporated into beef patties enhanced its physicochemical and sensory traits. Beyond its nutritional benefits, SCG‐derived phytochemicals demonstrated promising pharmacokinetic properties, with no inhibitory effects on cytochrome P450 enzymes. Additionally, molecular docking results confirmed strong binding interactions with the 2FLU (Kelch‐Neh2 Complex) target protein, particularly for chlorogenic acid, which exhibited the highest affinity. This clearly indicates that spent coffee grounds (SCG) can be incorporated into various meat products, offering advantages for both the industry and consumers by providing functional foods, as well as benefiting the environment by utilizing waste or by‐products. Valorization of SCG as an antioxidant and dietary fiber source in meat products is a suitable, natural, and cheap choice for the meat industry.

## Author Contributions

**Nazik Meziyet Dilek:** conceptualization (lead), data curation (lead), formal analysis (lead), funding acquisition (lead), investigation (lead), methodology (lead), project administration (lead), resources (lead), software (lead), supervision (lead), validation (lead), visualization (lead), writing – original draft (lead), writing – review and editing (lead).

## Conflicts of Interest

The author declares no conflicts of interest.

## Data Availability

This article includes all the datasets generated or analyzed during this study.
